# Comparative efficacy and safety of direct oral anticoagulants versus warfarin in non-valvular atrial fibrillation patients with adult congenital heart disease. Contemporary real-world propensity-matched retrospective cohort study

**DOI:** 10.1016/j.ijcchd.2026.100691

**Published:** 2026-06-24

**Authors:** Dennis D. Kumi, George Blankson, Irene Animah Acheampong, Samuel Michael Odoi, Sudhir Mungee, Timir Baman, Amit Mehrotra, Siddharth Shah

**Affiliations:** a Department of Medicine, University of Illinois College of Medicine at Peoria, Illinois, USA; b Department of Medicine, University Hospitals, Case Western Reserve University, Ohio, USA; c Department of Medicine, Clinical Research Unit, Carilion Clinic, Virginia Tech School of Medicine, USA; d Department of Medicine, John H. Stroger Hospital of Cook County Health, USA

## Abstract

**Background:**

Adults with congenital heart disease (ACHD) have a rising prevalence of atrial fibrillation/flutter (AF) and elevated thrombotic and bleeding risk. Although direct oral anticoagulants (DOACs) are established in non-ACHD AF, comparative effectiveness in ACHD is not well defined.

**Methods:**

In a propensity score–matched retrospective cohort study using the TriNetX database, adults (age ≥18) with ACHD and AF receiving oral anticoagulation were identified, excluding those with mechanical valves, LVAD, antiphospholipid syndrome, or rheumatic mitral stenosis. Patients were matched 1:1 to DOAC or warfarin. The primary outcomes were composite embolic events, composite bleeding events, and net clinical outcome (embolism, bleeding, or all-cause mortality). Secondary outcomes included gastrointestinal bleeding, nontraumatic intracranial hemorrhage (ICH), cardioembolic stroke, systemic arterial embolization, cerebral infarction, and all-cause mortality. Associations were estimated using hazard ratios (HRs). Sensitivity analysis was performed to confirm the robustness of magnitude and direction of outcomes.

**Results:**

After matching, 27,166 patients were included (13,583 per group). DOAC use was associated with lower hazard of net clinical outcome (HR 0.87, 95% CI 0.81–0.93), driven by reduced composite bleeding (HR 0.82, 95% CI 0.75–0.90) and all-cause mortality (HR 0.68, 95% CI 0.64–0.72). Hazard of composite embolic events did not differ (HR 0.94, 95% CI 0.87–1.03). DOACs were associated with lower hazard of systemic arterial embolization (HR 0.62, 95% CI 0.52–0.73), nontraumatic ICH (HR 0.74, 95% CI 0.61–0.90), and gastrointestinal bleeding (HR 0.84, 95% CI 0.76–0.93). No significant differences were observed for cerebral infarction or cardioembolic stroke.

**Conclusions:**

In a large propensity-matched cohort of patients with ACHD and AF, DOAC therapy was associated with lower hazard of net clinical outcome, all bleeding events, and all-cause mortality compared with warfarin, without increased embolic risk.

## Abbreviations

DOAC: Direct oral anticoagulant
AF: atrial fibrillation or flutter
ACHD: Adult congenital heart disease

**Clinical Perspective**

• These findings support the use of DOACs in ACHD-associated AF.
• Prospective studies are warranted to inform guideline-directed anticoagulation in this population.

**CRediT authorship contribution statement**

**Dennis D. Kumi:** Conceptualization, Data curation, Formal analysis, Methodology, Visualization, Writing – original draft. **George Blankson:** Conceptualization, Data curation, Formal analysis, Investigation, Writing – original draft, Writing – review & editing. **Irene Animah Acheampong:** Conceptualization, Data curation, Formal analysis, Investigation, Methodology, Project administration. **Samuel Michael Odoi:** Formal analysis, Methodology, Writing – original draft. **Sudhir Mungee:** Conceptualization, Supervision, Writing – review & editing. **Timir Baman:** Supervision, Writing – review & editing. **Amit Mehrotra:** Supervision, Writing – review & editing. **Siddharth Shah:** Conceptualization, Formal analysis, Supervision, Writing – original draft, Writing – review & editing.

### 1 Introduction

Improved survival after congenital heart disease repair has resulted in a growing population of adults with congenital heart disease (ACHD), now exceeding pediatric prevalence in developed countries [[1], [2], [3]]. Atrial fibrillation (AF) and flutter occur at younger ages in ACHD, driven by increased atrial scarring, chronic pressure and volume overload, cyanosis, and prior surgery [4]. Concomitant higher burden of cardiometabolic risk further amplifies arrhythmic burden in ACHD patients [5].

Additionally, AF in ACHD confers elevated thromboembolic risk, including in patients with low CHA_2_DS_2_-VASc scores [6,7]. Bleeding risk is also increased, and this may theoretically be heightened by prevalence of vascular malformations, hepatic dysfunction, and surgical complexity [8]. These competing risks complicate anticoagulation selection. Vitamin K antagonists have historically been used because ACHD patients were excluded from pivotal DOAC trials [[9], [10], [11]]. In non-ACHD AF, DOACs demonstrate non-inferior and at times superior efficacy with reduced intracranial hemorrhage [12]. However, altered anatomy and hepatic physiology, particularly in complex lesions such as Fontan circulation, may affect DOAC pharmacokinetics. There is currently no large high-level data on efficacy and safety of DOAC in ACHD -related AF populations.

Additionally, contemporary real-world comparative data in ACHD-specific AF cohorts remain limited. We therefore evaluated the comparative effectiveness and safety of DOAC versus warfarin in ACHD-associated AF using a large propensity-matched retrospective cohort.

### 2 Methodology

**Data source**: Data was obtained from the TriNetX Analytics Network database. TriNetX is a largely U.S.-based multicenter federated health research network aggregating anonymized data from electronic health records of more than 139 million patients at the time of our search and from more than 108 U S. health care organizations. Although the data are organized in an aggregate deidentified form, built-in analytics allow for the generation of patient-level data for cohort selection and matching, analyzing the incidence and prevalence of events in a cohort, and comparing characteristics and outcomes over time between matched cohorts at the patient level. TriNetX provides data, including demographics, diagnostic and procedural information, and standard measurements (including vital signs, laboratory results, and medications) using standardized coding systems (International Classification of Diseases-10th Revision-Clinical Modification [ICD-10 CM] and Current Procedural Terminology codes for diagnoses and procedures [CPT Codes], Logical Observation Identifiers names [LOIN] and codes for vital signs and laboratory values, and Rx Norm for medications). Because data are de-identified, this study was considered exempt from institutional review board oversight in accordance with institutional and federal guidelines. More information on the database can be found elsewhere [13].

**Study design**: In this propensity-match retrospective cohort study, we employed an incident user design [14]. By this design, we simulated the prospective nature of a randomized study to evaluate outcomes following initiation of DOAC or warfarin within the 2 cohorts. We set our index event to be new initiation of DOAC or warfarin and this reduced immortal-time bias as well as confounding from prior exposure [15]. We censored any outcome present prior to or on the day of index event, hence allowing an imprecise yet reasonable estimation of causal effect.

#### 2.1 Study population and cohort definition

Adult patients (Age ≥ 18 years) with adult congenital heart disease (ACHD) and atrial fibrillation/flutter (AF) were identified within the TriNetX network. Supplementary Table 1 provides details of various congenital defects included in our population. From this population, patients were classified into two exposure cohorts based on oral anticoagulant use: those treated with warfarin and those treated with direct oral anticoagulants (DOACs). Patients with Rheumatic mitral stenosis, Left Assisted Ventricular Device (LAVD), presence of mechanical valve, antiphospholipid syndrome, and those on both warfarin and a DOAC at or before index date were excluded from both cohorts. This was done to remove any compelling indication for warfarin therapy. We also excluded those with outcomes prior to index date. All search terms used to define the cohorts are provided in Supplementary Table 1 and Supplementary Table 2 in the appendix section.

#### 2.2 Index event and follow-up

The index event was defined as the first documented exposure to either warfarin or a direct oral anticoagulant. Patients were followed for up to five years (1825 days) after the index event. Outcomes were assessed beginning one day after the index event. Events documented prior to or on the day of the index event were excluded from outcome analyses to capture post-exposure outcomes only (incident outcomes).

#### 2.3 Outcomes

**The Primary outcomes** of our study were (1) composite-embolic-event (cardioembolic stroke, ischemic strokes or arterial embolization), (2) composite-bleeding-event (non-traumatic intracerebral hemorrhage or gastrointestinal hemorrhage) and (3) Net Clinical Outcome (Composite-embolic-event or composite-bleeding-event or all-cause mortality).

**Secondary outcomes** included rate of cardioembolic stroke, rate of ischemic stroke, rate of arterial embolization, rate of non-traumatic intracerebral hemorrhage, rate of gastrointestinal hemorrhage and rate of all-cause mortality. All outcomes were evaluated as incident events occurring after the index event, with pre-index events excluded as described above.

#### 2.4 Subgroup analysis

A prespecified subgroup analysis was performed to evaluate the consistency of treatment effects across varying levels of congenital heart disease complexity and physiologic risk. Following propensity score matching, patients were stratified into low-intermediate risk and high-risk ACHD groups according to the 2018 AHA/ACC Adult Congenital Heart Disease classification framework. The low-intermediate risk subgroup comprised patients with simple or moderate anatomic complexity lesions who lacked physiologic high-risk features. The high-risk subgroup included patients with great-complexity ACHD lesions and those with physiologic high-risk characteristics regardless of anatomic complexity. Physiologic high-risk features included; Fontan circulation, Eisenmenger physiology or cyanotic congenital heart disease. [16]. Within each subgroup, clinical outcomes were compared between the DOAC and warfarin cohorts using the same time-to-event analytical approach employed in the primary analysis. Hazard ratios with corresponding 95% confidence intervals were calculated for all efficacy and safety endpoints. Outcome frequencies, effect directionality, and magnitude of treatment effect were assessed within each risk stratum and compared with findings from the overall propensity-matched cohort to determine whether the observed associations between anticoagulant strategy and clinical outcomes were preserved across differing levels of ACHD risk.

#### 2.5 Propensity score matching

**Supplementary Fig. 2:**
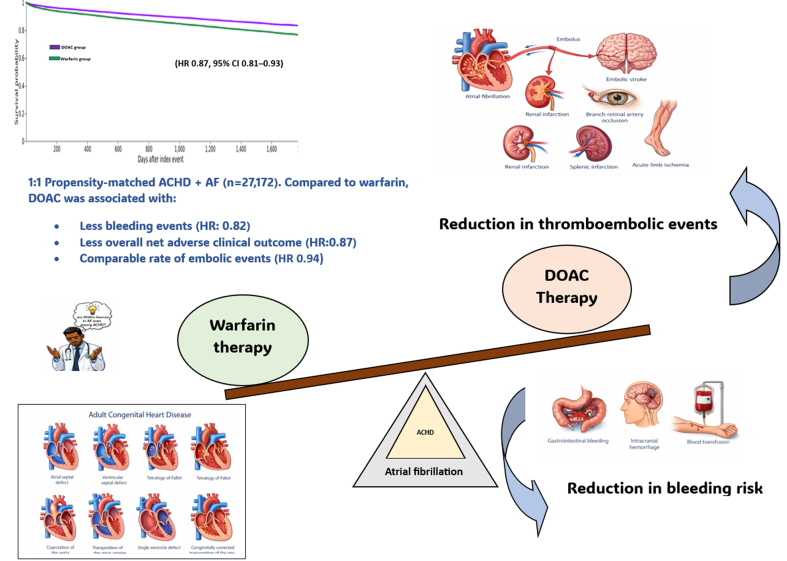
Graph and table of DOAC cohort versus Warfarin cohort before and after propensity matching. **DOAC**: direct oral anticoagulant. **Afib**: atrial fibrillation. **ACHD**: adult congenital heart disease.

To reduce confounding and improve comparability between exposure cohorts, propensity score matching was performed. Propensity scores were estimated using baseline demographic variables, including age, sex, race, and race/ethnicity, together with clinically relevant comorbidities captured at baseline. Patients in the DOAC cohort were matched 1:1 to patients in the warfarin cohort using nearest-neighbor matching. Covariate balance after matching was assessed using standardized mean differences and P values, with standardized mean differences below 0.10 considered indicative of adequate balance between cohorts. Supplementary Fig. 2 in the appendix shows details on propensity match curves and table.

#### 2.6 Statistical analysis and follow-up

Baseline characteristics were summarized and compared between matched cohorts using standardized mean differences, as appropriate. Clinical outcomes were evaluated using two approaches. First, risk-based analyses were conducted to estimate risk ratio with corresponding 95% confidence intervals. Second, time-to-event analyses were performed using Kaplan–Meier methods with log-rank testing, and hazard ratios were estimated using Cox proportional hazards regression models. All statistical tests were two-sided, and statistical significance was defined as a P value less than 0.05. All analyses were performed using the built-in analytical tools within the TriNetX platform. Cohorts were followed up after index event until death, outcome occurrence or end of study period. Median follow up was calculated using the reverse Kaplan Meier method and reported as median with interquartile ranges.

**Sensitivity Analyses**: To assess the robustness of our findings, we performed multiple sensitivity analyses. We performed a 30-day landmark analysis to eliminate guarantee-time bias and immortal-time bias and confirmed that all primary outcomes maintained their directionality and magnitude [17]. We also performed a negative control outcomes analysis to assess unmeasured confounding using acute cholecystitis and lung cancer, two conditions unrelated to anticoagulation choices. In this analysis, we concluded that there were no significant differences between DOAC and warfarin in rates of acute cholecystitis (0.7% vs 0.8%; HR 1.046, 95% CI 0.788–1.389) or lung cancer (1.1% vs 1.1%; HR 1.105, 95% CI 0.874–1.397), supporting the specificity of the observed associations in our study [18]. Finally, we performed a drug-specific subgroup analysis by varying our exposure from a DOAC (apixaban, dabigatran, rivaroxaban and edoxaban) to an apixaban-specific propensity matched cohort. This analysis led to similar primary outcomes as our composite DOAC group when compared to warfarin. This is a test confirming internal consistency of our exposure groups and further strengthening the robustness of our results. Details of sensitivity analysis are summarized in supplementary table 3.

**Missing Data**: Variables with incomplete ascertainment, particularly race and ethnicity, were handled using missing or unknown categories.

**Reporting Standards**: The study was designed and reported in accordance with the STROBE guidelines for observational research using routinely collected health data [19].

**Ethical considerations**: the study used a de-identified data source and poses no direct or indirect risks to patients. No institutional IRB approval was required per protocol.

### 3 Results

**Fig. 1:**
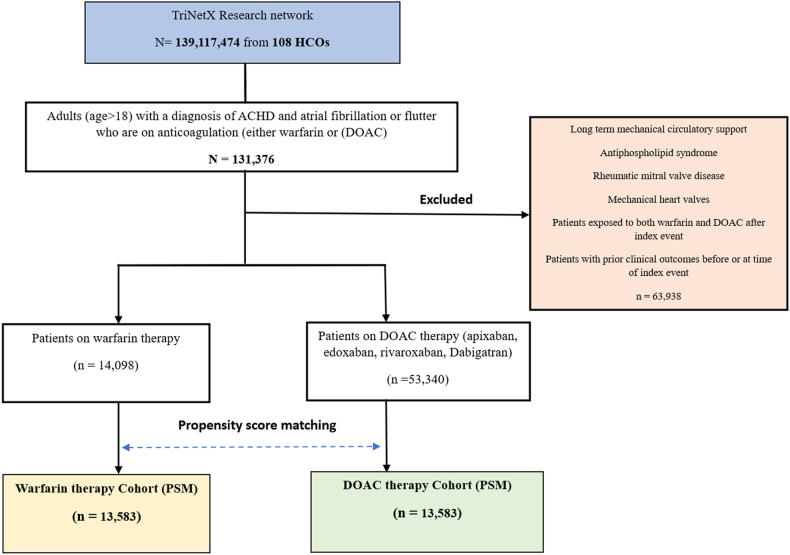
Flow diagram Flow diagram depicting inclusion and exclusion criteria for our study. **HCO** = Healthcare Organizations, **ACHD**: adult congenital heart disease.

Following 1:1 propensity score matching, 13,583 patients were included in each cohort (total n = 27,166). Fig. 1 shows a flow chart depicting study population and inclusion. Low-intermediate risk ACHD constituted 84.0% of the study population, compared with 16.0% classified as high risk. Distribution was balanced between the warfarin and DOAC cohorts (84.3% vs 83.6% low-intermediate risk; 15.7% vs 16.4% high risk, respectively). Atrial septal defect (32.0%) and ventricular septal defect (18.7%) were the most prevalent congenital lesions. Single ventricle/Fontan physiology was present in 2.0% of patients. Eisenmenger syndrome and truncus arteriosus communis were the least common diagnoses (0.1% each). Detailed lesion distribution is provided in Table 1. Within the propensity-matched DOAC cohort, apixaban was the predominant anticoagulant, accounting for 81.6% of patients, followed by rivaroxaban (15.8%), dabigatran (2.1%), and edoxaban (0.4%). Accordingly, the observed treatment effects within the DOAC arm were driven primarily by apixaban-treated patients. A detailed breakdown of oral anticoagulant utilization is provided in Supplementary table 4.

**Table 1:** Distribution of Propensity matched Cohort with ACHD-associated atrial fibrillation/flutter by Anatomic Complexity Class and Physiologic Qualifiers.

Defect/Condition	Total (n = 27,166)	Warfarin (n = 13,583)	DOAC (n = 13,583)
**LOW-INTERMEDIATE RISK ACHD (Anatomic Simple or Moderate Complexity Without Physiologic High-Risk Qualifiers)**			
**Anatomic class I — Simple complexity**			
Atrial Septal Defect (ASD)	8699 (32.0%)	4416 (32.5%)	4283 (31.5%)
Ventricular Septal Defect (VSD)	5088 (18.7%)	2519 (18.5%)	2569 (18.9%)
Patent Ductus Arteriosus (PDA)	3057 (11.3%)	1536 (11.3%)	1521 (11.2%)
Pulmonary Valve Stenosis	1178 (4.3%)	586 (4.3%)	592 (4.4%)
Aortic Valve Stenosis	899 (3.3%)	454 (3.3%)	445 (3.3%)
**Anatomic class II — Moderate complexity**			
Atrioventricular Septal Defect (AVSD)	962 (3.5%)	485 (3.6%)	477 (3.5%)
Tetralogy of Fallot (ToF)	937 (3.4%)	466 (3.4%)	471 (3.5%)
Coarctation of the Aorta	1152 (4.2%)	573 (4.2%)	579 (4.3%)
Ebstein Anomaly of the Tricuspid Valve	168 (0.6%)	85 (0.6%)	83 (0.6%)
Partial Anomalous Pulmonary Venous Connection (PAPVC)	96 (0.4%)	48 (0.4%)	48 (0.4%)
Pulmonary Artery Stenosis	580 (2.1%)	287 (2.1%)	293 (2.2%)
**Total (Low-Intermediate Risk ACHD)**	**22,816 (84.0%)**	**11,455 (84.3%)**	**11,361 (83.6%)**
**HIGH-RISK ACHD (Anatomic Great Complexity or Physiologic High-Risk Qualifiers)**			
**Anatomic class III — Great complexity**			
Single Ventricle/Fontan Physiology	544 (2.0%)	274 (2.0%)	270 (2.0%)
Transposition of the Great Arteries (TGA)	119 (0.4%)	60 (0.4%)	59 (0.4%)
Truncus Arteriosus Communis	28 (0.1%)	14 (0.1%)	14 (0.1%)
Total Anomalous Pulmonary Venous Connection (TAPVC)	72 (0.3%)	36 (0.3%)	36 (0.3%)
Double-Outlet Right Ventricle (DORV)	72 (0.3%)	36 (0.3%)	36 (0.3%)
Interrupted Aortic Arch (IAA)	36 (0.1%)	18 (0.1%)	18 (0.1%)
Pulmonary Atresia	678 (2.5%)	339 (2.5%)	339 (2.5%)
**Physiologic high-risk qualifiers**			
Cyanotic Congenital Heart Disease	4516 (16.6%)	2258 (16.6%)	2258 (16.6%)
Eisenmenger Syndrome	28 (0.1%)	14 (0.1%)	14 (0.1%)
**Total (High-Risk ACHD)**	**4350 (16.0%)**	**2128 (15.7%)**	**2222 (16.4%)**

**ACHD:** Adult congenital heart disease.

Median follow-up after matching was 1199 days (IQR 1359) in the DOAC cohort and 1825 days (IQR 1076) in the warfarin cohort.

As shown in Table 2, baseline characteristics were well matched between the two cohorts. Average age was 62 years with a male predominance of 60% and no significant residual differences across key demographic characteristics and comorbidities.

**Table 2:** Baseline Characteristics before and after propensity matching.

Characteristic	Before propensity score matching			After propensity score matching		
	DOAC (N = 53,340)	Warfarin (N = 14,098)	Std diff	DOAC (N = 13,583)	Warfarin (N = 13,583)	Std diff
Age at index (years)	66.5 ± 12.5	62.1 ± 13.8	0.332	62.4 ± 14.2	62.2 ± 13.7	0.016
Female	22,503 (42.2%)	5437 (39.9%)	0.047	5360 (39.5%)	5420 (39.9%)	0.009
Male	30,815 (57.8%)	8191 (60.1%)	0.047	8217 (60.5%)	8156 (60.0%)	0.009
White	42,255 (79.2%)	10,510 (77.1%)	0.052	10,650 (78.4%)	10,483 (77.2%)	0.030
Black or African American	5410 (10.1%)	1460 (10.7%)	0.018	1386 (10.2%)	1449 (10.7%)	0.015
Asian	1826 (3.4%)	467 (3.4%)	<0.001	400 (2.9%)	464 (3.4%)	0.027
American Indian or Alaska Native	174 (0.3%)	52 (0.4%)	0.009	44 (0.3%)	52 (0.4%)	0.010
Native Hawaiian or Other Pacific Islander	385 (0.7%)	83 (0.6%)	0.014	70 (0.5%)	83 (0.6%)	0.013
Other race	919 (1.7%)	193 (1.4%)	0.025	185 (1.4%)	193 (1.4%)	0.005
Unknown race	2371 (4.4%)	870 (6.4%)	0.086	848 (6.2%)	859 (6.3%)	0.003
Hypertension	33,257 (62.3%)	7303 (53.6%)	0.179	7181 (52.9%)	7282 (53.6%)	0.015
Hyperlipidemia	29,802 (55.9%)	5901 (43.3%)	0.254	5863 (43.2%)	5896 (43.4%)	0.005
Coronary artery disease	19,078 (35.8%)	4694 (34.4%)	0.028	4515 (33.2%)	4674 (34.4%)	0.025
Type 2 diabetes mellitus	14,045 (26.3%)	3382 (24.8%)	0.035	3295 (24.3%)	3372 (24.8%)	0.013
Systolic heart failure	7370 (13.8%)	2017 (14.8%)	0.028	1912 (14.1%)	2002 (14.7%)	0.019
Chronic kidney disease	9343 (17.5%)	2457 (18.0%)	0.013	2206 (16.2%)	2443 (18.0%)	0.046
Sleep apnea	10,634 (19.9%)	1922 (14.1%)	0.156	1781 (13.1%)	1919 (14.1%)	0.030
COPD	7180 (13.5%)	1533 (11.2%)	0.067	1425 (10.5%)	1528 (11.2%)	0.024
TIA	3528 (6.6%)	636 (4.7%)	0.085	511 (3.8%)	635 (4.7%)	0.045
Smoking	12,351 (23.2%)	1881 (13.8%)	0.243	1681 (12.4%)	1880 (13.8%)	0.043
Primary pulmonary hypertension	517 (1.0%)	330 (2.4%)	0.113	254 (1.9%)	297 (2.2%)	0.022
Secondary pulmonary hypertension	6287 (11.8%)	1901 (13.9%)	0.064	1723 (12.7%)	1867 (13.7%)	0.031
Obesity or Overweight	11, 544 (21.6%)	4, 989 (35.4%)	0.612	4761 (35.1%)	4798 (35.3%)	0.027
Liver disease	4581 (8.6%)	893 (6.5%)	0.077	778 (5.7%)	892 (6.6%)	0.035
Aspirin use	24,333 (45.6%)	5526 (40.5%)	0.103	5331 (39.2%)	5507 (40.5%)	0.026
P2Y12 inhibitor use	7759 (14.5%)	1367 (10.0%)	0.138	1275 (9.4%)	1367 (10.1%)	0.023

Table 2 summarizes the baseline characteristics of both the unmatched cohort as well as the cohorts after propensity score matching. **PSM**: Propensity score matching. **TIA**: Transient ischemia attack, **std diff**: standardized mean difference. Values are mean ± SD or n (%). Any characteristic with a standardized mean difference between cohorts <0.10 is considered to be well matched.

#### 3.1 Outcomes

**Fig. 2:**
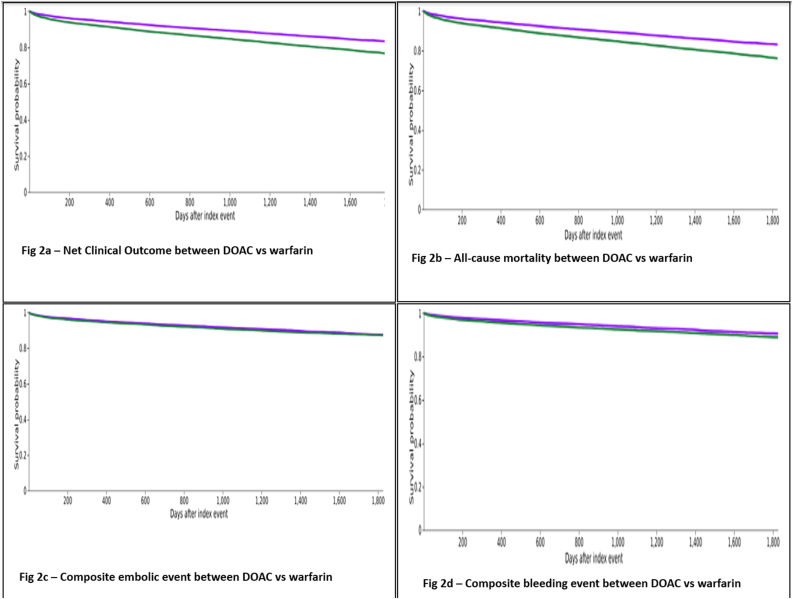
Kaplan–Meier curves showing Clinical outcome after propensity score matching comparing Atrial fibrillation or flutter patients with ACHD on DOAC vs Warfarin therapy. **ACHD**: Adult congenital heart disease, **DOAC**: direct oral anticoagulant.

**Primary Outcomes**: Net clinical outcome occurred in 13.2% of DOAC-treated patients versus 16.7% with warfarin and was associated with a 13% lower hazard under DOAC therapy (HR 0.87, 95% CI 0.81–0.93). Likewise, Composite bleeding occurred in 6.4% versus 8.7% and was associated with an 18% hazard reduction with DOAC use (HR 0.82, 95% CI 0.75–0.90). However, Composite embolic events occurred in 8.7% versus 10.3%, respectively, without statistically significant difference in hazard (HR 0.94, 95% CI 0.87–1.03). Fig. 2 below depicts Kaplan-Meier curves of primary outcome events between the 2 cohorts. Further details are as shown in Table 3 below.

**Table 3:** Clinical outcomes in propensity-matched cohorts (DOAC vs Warfarin).

Outcome	DOAC Group $\frac{\mathrm{Event}\;\mathrm{frequency}}{\mathrm{Total}\;\mathrm{in}\;\mathrm{cohort}}$ ($\frac{n}{N}$)%	Warfarin Group $\frac{\mathrm{Event}\;f\mathrm{requency}}{\mathrm{Total}\;\mathrm{in}\;\mathrm{cohort}}$ ($\frac{n}{N}$)%	Hazard Ratio (95% CI)
**PRIMARY OUTCOMES**			
Net Clinical Outcome	$\frac{1,471\;}{11,141}$ (13.2%)	$\frac{1,817\;}{10,873}$ (16.7%)	0.87 (0.81–0.93)
Composite Embolic Event	$\frac{1,005\;}{11,515}$ (8.7%)	$\frac{1,166}{11,331}$ (10.3%)	0.94 (0.87–1.03)
Composite Bleeding Event	$\frac{824}{12,965}$ (6.4%)	$\frac{1,116}{12,835}$ (8.7%)	0.82 (0.75–0.90)
**SECONDARY OUTCOMES**			
All-cause Mortality	$\frac{1,620}{13,583}$ (11.9%)	$\frac{2,716}{13,583}$ (20.0%)	0.68 (0.64–0.72)
Cerebral Infarction	$\frac{881}{11,681}$ (7.5%)	$\frac{962}{11,674}$ (8.2%)	1.03 (0.94–1.13)
Cardioembolic Stroke	$\frac{317}{13,038}$ (2.4%)	$\frac{315}{13,006}$ (2.4%)	1.11 (0.95–1.30)
Systemic Arterial Embolism	$\frac{222}{13,333}$ (1.7%)	$\frac{390}{13,126}$ (3.0%)	0.62 (0.52–0.73)
Non-traumatic ICH	$\frac{165}{13,396}$ (1.2%)	$\frac{256}{13,390}$ (1.9%)	0.74 (0.61–0.90)
GI Bleed	$\frac{690}{13,137}$ (5.3%)	$\frac{914}{13,017}$ (7.0%)	0.84 (0.76–0.93)

Table 3: Table of clinical adverse events frequency and Hazard ratio comparing atrial fibrillation patients with ACHD on DOAC therapy vs warfarin therapy. **(**$\frac{n}{N}$**)%** represents the relative frequency of each outcome**.**n = the frequency of each outcome**. N =** the total number in each cohort excluding those who already had that specified outcome at index date of being started on either DOAC or Warfarin.

**DOAC**: Direct oral anticoagulant. **GI**: gastrointestinal, **ICH**: intracerebral hemorrhage. **Net Clinical Outcome** (composite thrombotic event or composite hemorrhagic event or all-cause mortality.

**Fig. 3:**
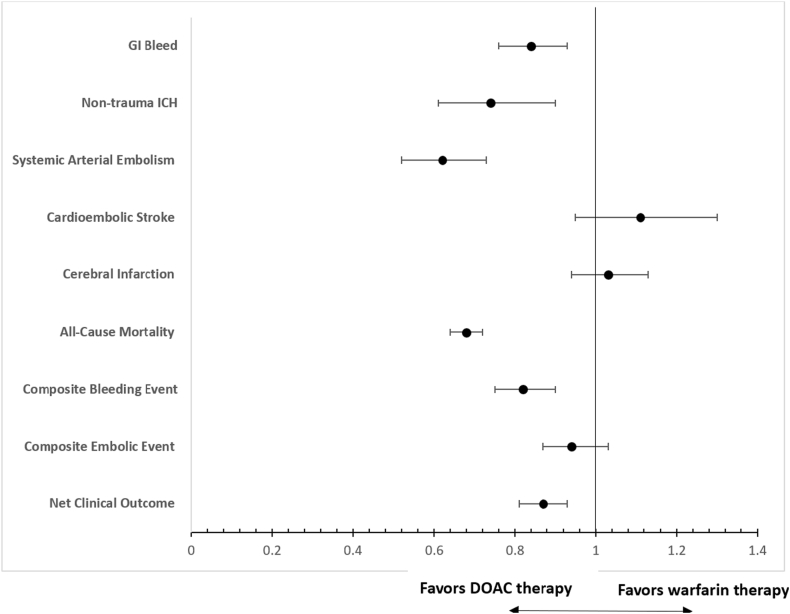
Forest plot showing hazard rations and 95% confidence intervals for various primary and secondary outcomes of DOAC vs warfarin therapy in ACHD-associated atrial fibrillation **ACHD**: Adult congenital heart disease, **DOAC**: direct oral anticoagulant.

**Secondary Outcomes:** All-cause mortality occurred in 11.9% of DOAC-treated patients versus 20.0% with warfarin and was associated with a 32% lower hazard under DOAC therapy (HR 0.68, 95% CI 0.64–0.72). Cerebral infarction occurred in 7.5% versus 8.2%, without significant difference in hazard (HR 1.03, 95% CI 0.94–1.13). Cardioembolic stroke occurred in 2.4% in both groups, with no statistically significant hazard difference (HR 1.11, 95% CI 0.95–1.30). Arterial embolism occurred in 1.7% versus 3.0% and was associated with a 38% lower hazard with DOAC therapy (HR 0.62, 95% CI 0.52–0.73). Intracerebral hemorrhage occurred in 1.2% versus 1.9% and was associated with a 26% lower hazard (HR 0.74, 95% CI 0.61–0.90). Gastrointestinal bleeding occurred in 5.3% versus 7.0% and was associated with a 16% lower hazard under DOAC use (HR 0.84, 95% CI 0.76–0.93). Fig. 3 below depicts a forest plot to graphically display the hazard ratios of secondary outcomes. Further details are as shown in Table 3 below.

#### 3.2 Sub-group analysis

**Fig. 4:**
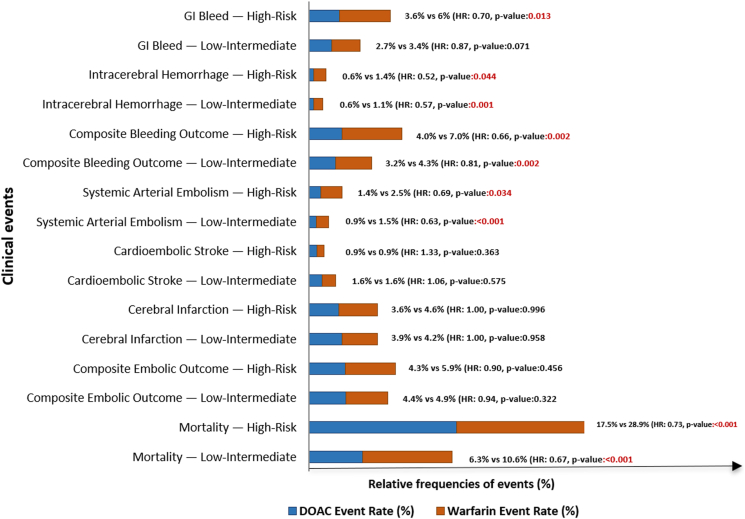
Composite bar graph showing pre-specified subgroup analysis comparing clinical outcomes of ACHD-associated atrial fibrillation between DOAC and warfarin based on ACHD risk stratification. **ACHD**: Adult congenital heart disease, **DOAC**: direct oral anticoagulant.

In a pre-specified subgroup analysis, patients were stratified into low-intermediate risk ACHD and high-risk ACHD. Overall, treatment effects were consistent with those observed in the overall cohort. Among low-intermediate risk ACHD patients, DOAC therapy was associated with lower mortality (5.1% vs 8.6%; HR 0.67, p < 0.001), systemic arterial embolism (0.9% vs 1.5%; HR 0.63, p < 0.001), composite bleeding (3.2% vs 4.3%; HR 0.81, p = 0.002), and intracerebral hemorrhage (0.6% vs 1.1%; HR 0.57, p < 0.001). No significant differences were observed for composite embolic events, cerebral infarction, cardioembolic stroke, or gastrointestinal bleeding. Among high-risk ACHD patients, DOAC therapy remained associated with lower mortality (8.9% vs 13.8%; HR 0.73, p < 0.001), composite bleeding (4.0% vs 7.0%; HR 0.66, p = 0.002), intracerebral hemorrhage (0.6% vs 1.4%; HR 0.52, p = 0.044), and gastrointestinal bleeding (3.6% vs 5.8%; HR 0.70, p = 0.013). No significant differences were observed for embolic outcomes. These findings demonstrate preservation of the mortality and bleeding benefits of DOAC therapy across ACHD risk strata, with the greatest and most consistent effects observed for major bleeding outcomes. Details of secondary analysis are depicted in Fig. 4 below. Supplementary Table 5 shows details of outcomes of subgroup analysis.

### 4 Discussion

In this AF-specific ACHD cohort, key efficacy outcomes including composite embolic events (8.7% vs 10.3%; HR 0.94, 95% CI 0.87–1.03), cerebral infarction (7.5% vs 8.2%; HR 1.03, 95% CI 0.94–1.13) and cardioembolic stroke (2.4% vs 2.4%; HR 1.11, 95% CI 0.95–1.30) were comparable between warfarin and DOAC whereas systemic arterial embolism was lower with DOAC (1.7% vs 3.0; HR 0.62, 95% CI 0.52–0.73). In non-ACHD AF, DOACs demonstrated at least noninferior and in some trials superior efficacy compared with warfarin [11,20,21]. Our findings are reflective of this well-known fact. Smaller ACHD series as well as metanalysis have reported comparable thromboembolic outcomes between strategies [[22], [23], [24], [25]]. In a large administrative analysis of over 4400 ACHD that included mixed anticoagulation indications besides atrial fibrillation, as well as similar smaller studies, DOACs were reported to have high rates of embolic events [26,27]. This is likely reflective of heterogeneity. The overall population after propensity matching included only 16% of high risk ACHD anatomy and physiology. The direction and magnitude of effect for efficacy, bleeding, mortality, and net clinical outcomes were preserved across different risk strata (high-risk ACHD vs low-intermediate risk ACHD) in our pre-specified subgroup analysis, suggesting that the observed associations are broadly applicable throughout the ACHD spectrum rather than being confined to lower-risk patients.

**Safety outcomes** consistently favored DOAC therapy including composite bleeding events (6.4% vs 8.7; HR 0.82, 95% CI 0.75–0.90), non-traumatic intracerebral hemorrhage (1.2% vs 1.9%; HR 0.74, 95% CI 0.61–0.90), and gastrointestinal bleeding (5.3% vs 7.0%; HR 0.84, 95% CI 0.76–0.93). Reduced intracranial bleeding with DOACs is a consistent finding in large, randomized AF trials and meta-analyses [11,12]. The predictable pharmacokinetics and absence of labile anticoagulation likely mitigate supratherapeutic exposure observed with warfarin [10]. Unfortunately, available studies have been among patients with undifferentiated indications for anticoagulation including vascular thrombosis and have led to very conflicting results [24,25,28,29]. Our findings are consistent with studies from the international NOTE Registry, which demonstrated low rates of thromboembolic and major bleeding events among ACHD patients treated with NOACs [30,31]. The present study however directly compared propensity-matched DOAC and warfarin cohorts within an AF-specific ACHD population and demonstrated superior safety with preserved efficacy.

**All-cause mortality** was lower in DOAC group compared to warfarin (11.9% vs 20.0%; HR 0.68, 95% CI 0.64–0.72). While this may not deviate from mortality reduction with certain DOACs as has been observed in non-ACHD AF populations, causal association cannot be hypothesized since this study was not designed to accurately capture atrial fibrillation or anticoagulation related mortality per se [11,12]. There is possible residual confounding including preferential warfarin use in patients with greater anatomic complexity or hepatic dysfunction not fully captured in registry data. Importantly, propensity matching resulted in similar distributions of low-intermediate and high-risk ACHD patients between treatment groups, arguing against substantial bias from preferential allocation of higher-risk congenital lesions to warfarin. However, mortality findings should be interpreted cautiously. Physician prescribing preferences may have been influenced by factors not captured within the dataset, including frailty, occult bleeding risk, gastrointestinal vascular malformations, hepatic dysfunction, anticipated adherence and socioeconomic barriers including cost prohibition. Therefore, residual indication bias and unmeasured confounding cannot be completely excluded.

Overall atrial fibrillation-associated and anticoagulation-associated adverse outcomes was captured in the **Net clinical outcome** which was reduced in DOAC group compared to warfarin (13.2% vs 16.7%; HR 0.87, 95% CI 0.81–0.93). This composite outcome integrates ischemic and hemorrhagic risk and aligns directionally with prior AF data demonstrating favorable overall clinical profiles for DOACs [12]. In an AF-focused ACHD population, these findings support comparable thromboembolic protection with superior safety and lower mortality, yielding a favorable net clinical balance. In a small retrospective study of 139 ACHD patients, DOAC was noted to reduce clinical adverse outcomes of bleeding and thrombosis among high-risk patients with Fontan physiology [24].

#### 4.1 Strength of study

This study has several strengths. It represents a large, contemporary, multinational real-world cohort of adults with congenital heart disease and atrial fibrillation, a population historically underrepresented in randomized trials [10,11]. The AF-specific design reduces indication heterogeneity seen in prior mixed-cohort analyses [26]. Propensity score matching enhanced baseline comparability between treatment groups, mitigating measured confounding. Time-to-event analyses using hazard ratios allowed assessment of longitudinal risk rather than cross-sectional event frequency. Use of strong end points such as stroke events, bleeding events and mortality and avoidance of subjective or patient reported events improves specificity and reduces misclassification bias in retrospective research. Specific exclusion of preexisting outcomes at the time of index date allows for meaningful post-exposure outcome analysis. Exclusion of certain high thrombotic risk populations that are excluded in DOAC trials based on randomized data of inferiority of DOACS including LVAD, mechanical valves, rheumatic mitral stenosis and antiphospholipid syndrome remove some amount of confounding by indication. The study excluded DOAC-warfarin crossover to be able to adjudicate true impact of either therapy on safety or efficacy outcomes. Even though the overall propensity matched cohort was predominantly low-intermediate risk, prespecified subgroup analyses demonstrated consistent treatment effects across both low-intermediate and high-risk ACHD populations. The direction and magnitude of effect for efficacy, bleeding, mortality, and net clinical outcomes were preserved across risk strata, suggesting that the observed associations are broadly applicable throughout the ACHD spectrum rather than being confined to lower-risk patients.

#### 4.2 Limitations

This was a retrospective, EHR-based observational analysis, so causality cannot be established. Despite propensity score matching, residual confounding is likely, particularly from unmeasured factors such as congenital lesion complexity, surgical history, clinical severity, and prescribing bias. Medication exposure reflects prescriptions rather than confirmed adherence, and warfarin quality metrics (e.g., time in therapeutic range/INR control) were unavailable. Outcomes were identified using diagnosis codes without central adjudication, creating potential misclassification. Follow-up and data completeness may vary across health systems, which can affect generalizability. Our study did not separately study high risk ACHD such as those with blind loop end and slow flow states including Fontan physiology, atrial switch cases and single ventricle disorders. Our results may not be applied these populations. Although obesity and chronic kidney disease were broadly included in the propensity model, detailed measures such as body mass index, serum creatinine, creatinine clearance, and estimated glomerular filtration rate were unavailable. These factors influence anticoagulant dosing and are associated with bleeding and thromboembolic outcomes. Consequently, residual confounding related to anticoagulant dosing appropriateness cannot be excluded, particularly for bleeding outcomes.

### 5 Conclusions

In this large observational study of adults with congenital heart disease and non-valvular atrial fibrillation or flutter, DOAC treatment was associated with a significantly lower hazard of the net clinical outcomes mainly driven by a reduction in hazard of composite bleeding events and all-cause mortality without a statistically significant difference in efficacy outcomes including composite thrombotic events and cardioembolic strokes. Collectively, these findings support the hypothesis that treatment of atrial fibrillation in ACHD with DOAC offers non-inferior efficacy while offering superior safety as compared to warfarin. Large prospective studies will be required to confirm this hypothesis and to make strong recommendations for future guideline directed anticoagulation.

**Perspectives:** In a general population of ACHD with AF, DOAC in comparison with warfarin, provides similar thromboembolic efficacy while reducing bleeding events.

**Translational Outlook**: Further large database study or prospective research with emphasis on evaluating safety and efficacy of DOAC in high risk ACHD including Fontan physiology, single ventricle anatomy and post atrial switch anatomy patients are needed. At the moment, warfarin is still favored in these groups simply due to lack of sufficient data with DOAC.

### Disclosures

All authors of this study declare no conflict of interest pertaining to the conduct of this study or its publication.

### Funding

The authors of this manuscript did not receive any funding.

## Declaration of competing interest

The authors declare that they have no known competing financial interests or personal relationships that could have appeared to influence the work reported in this paper.

## Figures and Tables

**Supplementary Fig. 1ztarttnqtech:**
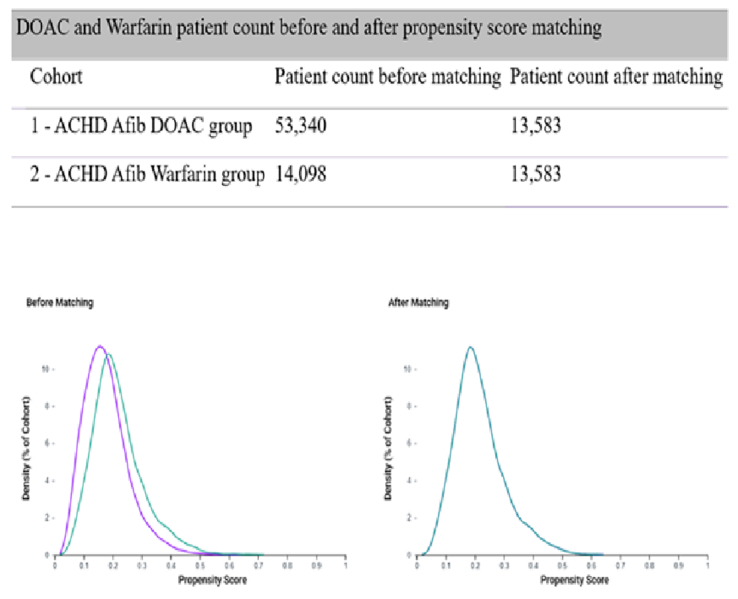
Visual Summary of study.
