# Supplementary material for: Comparative efficacy and safety of direct oral anticoagulants versus warfarin in non-valvular atrial fibrillation patients with adult congenital heart disease. Contemporary real-world propensity-matched retrospective cohort study
Source: Int J Cardiol Congenit Heart Dis. 2026 Jun 24;25:100691. doi: 10.1016/j.ijcchd.2026.100691 (PMC13393154; doi:10.1016/j.ijcchd.2026.100691)
Supplement: Multimedia component 1 [file mmc1.docx]

**Supplementary Figure 1**; Visual Summary of study

**Supplementary Figure 2**: Graph and table of DOAC cohort versus Warfarin cohort before and after propensity matching. **DOAC**: direct oral anticoagulant. **Afib**: atrial fibrillation. **ACHD**: adult congenital heart disease.

Before propensity score matching, the treatment and control groups demonstrated distinct and partially overlapping propensity score distributions, whereas after matching, the distributions were nearly identical, indicating improved covariate balance between the cohorts.

APPENDIX

**Supplementary Table 1. ACHD Diagnoses and Corresponding ICD-10-CM Codes by Risk Subgroup**

| **Defect / Condition** | **ICD-10-CM Code(s)** |
| --- | --- |
| **LOW-INTERMEDIATE RISK ACHD (Anatomic Simple or Moderate Complexity Without Physiologic High-Risk Qualifiers)** | |
| **ANATOMIC CLASS I — SIMPLE COMPLEXITY** | |
| Atrial Septal Defect (ASD) | Q21.1, Q21.11 |
| Ventricular Septal Defect (VSD) | Q21.0 |
| Patent Ductus Arteriosus (PDA) | Q25.0 |
| Pulmonary Valve Stenosis | Q22.1 |
| Aortic Valve Stenosis | Q23.0 |
| **ANATOMIC CLASS II — MODERATE COMPLEXITY** | |
| Atrioventricular Septal Defect (AVSD) | Q21.2, Q21.21, Q21.23 |
| Tetralogy of Fallot (ToF) | Q21.3 |
| Coarctation of the Aorta | Q25.1 |
| Ebstein Anomaly of the Tricuspid Valve | Q22.5 |
| Partial Anomalous Pulmonary Venous Connection (PAPVC) | Q26.3 |
| Pulmonary Artery Stenosis | Q25.79 |
| **HIGH-RISK ACHD (Anatomic Great Complexity or Physiologic High-Risk Qualifiers)** | |
| **ANATOMIC CLASS III — GREAT COMPLEXITY** | |
| Single Ventricle / Fontan Physiology | Q23.4, Q22.4, I27.84, I27.849 |
| Transposition of the Great Arteries (TGA) | Q20.3 |
| Truncus Arteriosus Communis | Q20.0 |
| Total Anomalous Pulmonary Venous Connection (TAPVC) | Q26.2 |
| Double-Outlet Right Ventricle (DORV) | Q20.1 |
| Interrupted Aortic Arch (IAA) | Q25.21 |
| Pulmonary Atresia | Q22.0, Q25.5 |
| **PHYSIOLOGIC HIGH-RISK QUALIFIERS** | |
| Cyanotic Congenital Heart Disease | Q24.9 |
| Eisenmenger Syndrome | I27.83 |

**Supplementary table 2. ICD and Medication Codes Used in Cohort Definition**

| Variables | ICD Code /PCS Code/ CPT Code |
| --- | --- |
| **Inclusion criteria variables** | |
| Atrial fibrillation or flutter | I48 |
| **Exclusion criteria variables** | |
| Presence of LVAD | Z95.811 |
| Rheumatic mitral valve disease | I05.0 |
| Presence of Prosthetic Heart Valve | Z95.2 |
| Antiphospholipid Syndrome | D68.61 |
| **Baseline Variables** | |
| Essential (primary) hypertension | I10 |
| Hyperlipidemia | E78 |
| Ischemic heart disease | I25 |
| Type 2 diabetes mellitus | E11 |
| Systolic (congestive) heart failure | I50.2 |
| Chronic kidney disease (CKD) | N18 |
| Sleep apnea | G47.3 |
| Chronic obstructive pulmonary disease, unspecified | J44.9 |
| Transient cerebral ischemic attacks and related syndromes | G45 |
| Personal history of nicotine dependence | Z87.891 |
| Pulmonary hypertension | I27.0, I27.2 |
| Liver disease | K76 |
| **Medications** | |
| Aspirin (RxNorm) | 1191 |
| Clopidogrel (RxNorm) | 32968 |
| warfarn(RxNorm) | 11289 |
| apixaban(RxNorm) | 1037042 |
| edoxaban(RxNorm) | 1599538 |
| rivaroxaban(RxNorm) | 1364430 |
| dabigatran(RxNorm) | 1114195 |
| **Outcome Variables** | |
| Cerebral Infarction/Stroke | I63 |
| Arterial Embolism and Thrombosis | I74 |
| Cardio-embolic stroke | I63.4 |
| Gastrointestinal Hemorrhage | K92.2 |
| Non traumatic Intracerebral Hemorrhage | I61 |

**Supplementary table 3:**Composite outcomes table showing sensitivity analysis for primary outcomes of atrial fibrillation with ACHD between DOAC group and warfarin group.

| **Outcome** | **Event rate with DOAC** $\frac{\boldsymbol{n}}{\boldsymbol{N}}$ **(%)** | **Event rate with warfarin** $\frac{\boldsymbol{n}}{\boldsymbol{N}}$ **(%)** | **Hazard Ratio (95% CI)** |
| --- | --- | --- | --- |
| **Sensitivity Analysis (30-Day Landmark): DOAC vs Warfarin** | | | |
| Net Clinical Outcome (Unnamed Composite) | $\frac{1,213}{10,520}$ (11.5%) | $\frac{1,555}{10,325}$ (15.1%) | 0.86 (0.79–0.92) |
| Embolic Outcome (Composite Embolic) | $\frac{837}{10,932}$ (7.7%) | $\frac{994}{10,817}$ (9.2%) | 0.94 (0.86–1.03) |
| Bleeding Outcome (Composite Bleeding) | $\frac{696}{12,440}$ (5.6%) | $\frac{965}{12,315}$ (7.8%) | 0.85 (0.74–0.90) |
| **Apixaban vs Warfarin Sensitivity Analysis (After Propensity Matching)** | | | |
| Net Clinical Outcome (Composite) | $\frac{830}{5,727}$ (14.5%) | $\frac{895}{5,433}$ (16.5%) | 0.771 (0.701–0.847) |
| Embolic Outcome (Composite Embolic) | $\frac{554}{5,937}$ (9.3%) | $\frac{579}{5,700}$ (10.2%) | 0.818 (0.728–0.919) |
| Bleeding Outcome (Composite Hemorrhage) | $\frac{494}{6,639}$ (7.4%) | $\frac{566}{6,543}$ (8.7%) | 0.760 (0.673–0.857) |
| **Negative Control Outcomes: DOAC vs Warfarin** | | | |
| Cholecystitis (Acute cholecystitis) | $\frac{91}{13,057}$ (0.7%) | $\frac{102}{13,085}$ (0.8%) | 1.046 (0.788–1.389) |
| Lung Cancer (Malignant neoplasm of bronchus and lung) | $\frac{137}{12,922}$ (1.1%) | $\frac{144}{12,993}$ (1.1%) | 1.105 (0.874–1.397) |

Table shows 30-day landmark analysis, one-way deterministic analysis using apixaban-specific group in place of DOAC-group as well as negative control outcome analysis using two unrelated outcomes including acute cholecystitis and Lung cancer.

**Supplementary table 4**. Relative frequencies of various Oral anticoagulants among adult congenital disease patients with atrial fibrillation/flutter.

| Oral anticoagulant type | Total number before Propensity Score Matching | Total number after Propensity Score Matching |
| --- | --- | --- |
| Apixaban | 43,550 | 11,089 |
| Rivaroxaban | 8,444 | 2150 |
| Edoxaban | 235 | 60 |
| Dabigatran | 1111 | 284 |
| Warfarin | 14,098 | 13583 |

**Supplementary table 5; ACHD-Associated AF: Clinical Outcomes by Risk Subgroup DOAC vs. Warfarin**

| **Clinical Outcome** | **LOW-INTERMEDIATE RISK ACHD (n = 22,816)** | | | |  | **HIGH-RISK ACHD (n = 4,350)** | | | |
| --- | --- | --- | --- | --- | --- | --- | --- | --- | --- |
|  | **DOAC n (%)** | **Warfarin n (%)** | **Hazard Ratio (95% CI)** | **p-value** |  | **DOAC n (%)** | **Warfarin n (%)** | **Hazard Ratio (95% CI)** | **p-value** |
| Mortality | 719 (6.3%) | 1,215 (10.6%) | **0.67 (0.61–0.74)** | **<0.001** |  | 388 (17.5%) | 615 (28.9%) | **0.73 (0.64–0.83)** | **<0.001** |
| Composite Embolic Outcome | 496 (4.4%) | 567 (4.9%) | 0.94 (0.83–1.06) | 0.322 |  | 96 (4.3%) | 126 (5.9%) | 0.90 (0.69–1.18) | 0.456 |
| Cerebral Infarction | 443 (3.9%) | 484 (4.2%) | 1.00 (0.88–1.14) | 0.958 |  | 79 (3.6%) | 97 (4.6%) | 1.00 (0.74–1.35) | 0.996 |
| Cardioembolic Stroke | 180 (1.6%) | 184 (1.6%) | 1.06 (0.86–1.30) | 0.575 |  | 21 (0.9%) | 19 (0.9%) | 1.33 (0.72–2.49) | 0.363 |
| Systemic Arterial Embolism | 98 (0.9%) | 170 (1.5%) | **0.63 (0.49–0.80)** | **<0.001** |  | 32 (1.4%) | 53 (2.5%) | 0.69 (0.44–0.99) | 0.034 |
| Composite Bleeding Outcome | 358 (3.2%) | 487 (4.3%) | **0.81 (0.71–0.93)** | **0.002** |  | 88 (4.0%) | 149 (7.0%) | **0.66 (0.51–0.86)** | **0.002** |
| Intracerebral Hemorrhage | 64 (0.6%) | 126 (1.1%) | **0.57 (0.42–0.77)** | **<0.001** |  | 13 (0.6%) | 30 (1.4%) | **0.52 (0.27–0.99)** | **0.044** |
| GI Bleed | 306 (2.7%) | 389 (3.4%) | 0.87 (0.75–1.01) | 0.071 |  | 80 (3.6%) | 128 (6.0%) | **0.70 (0.53–0.93)** | **0.013** |
